# Gene expression profile analysis of the rabbit retinal vein occlusion model

**DOI:** 10.1371/journal.pone.0236928

**Published:** 2020-07-31

**Authors:** Takuma Neo, Makoto Gozawa, Yoshihiro Takamura, Masaru Inatani, Masaya Oki

**Affiliations:** 1 Department of Applied Chemistry and Biotechnology, Graduate School of Engineering, University of Fukui, Fukui, Japan; 2 Department of Ophthalmology, Faculty of Medical Science, University of Fukui, Fukui, Japan; 3 Life Science innovation center, University of Fukui, Fukui, Japan; University of Florida, UNITED STATES

## Abstract

The rabbit retinal vein occlusion (RVO) model is an experimental system that mimics retinal ischemic diseases in humans. The rabbit RVO model is widely used to assess the therapeutic efficacy of various experimental surgical procedures. In the present study, we measured temporal retinal expression of *Vegfa*, which is known as an ischemic response gene, in rabbit RVO. This analysis revealed that the retinal *Vegfa* transcriptional response began 7 days after generation of RVO, rather than immediately after induction of ischemia. Next, in order to analyze ischemia-induced changes in gene expression profiles, we performed microarray analysis of day 7 RVO retina versus control retina. The angiogenic regulators *Dcn* and *Mmp1* and pro-inflammatory factors *Mmp12* and *Cxcl13* were significantly upregulated in RVO retinas. Further, we suggest that epigenetic regulation via the REST/cofactor-complex could contribute to RVO pathology. Among human homologous genes in rabbits, genes associated with hypoxia, angiogenesis, and inflammation were significantly upregulated in RVO retinas. Components of the Tumor necrosis factor-alpha (TNFα) and Nuclear factor-kappa B (NF-κB) pathways, which play regulatory roles in angiogenesis and inflammation, were significantly upregulated in RVO, and the expression levels of downstream factors, such as the transcription factor AP-1 and chemokines, were increased. Further, connectivity map analyses suggested that inhibitors of the NF-κB pathway are potential therapeutic agents for retinal ischemic disease. The present study revealed new insights into the pathology of retinal ischemia using the rabbit RVO model, which accurately recapitulates human disease.

## Introduction

Retinal ischemic diseases such as diabetic retinopathy and retinal vein occlusion (RVO) cause severe visual impairments, and are a leading cause of blindness [1, 2]. In the ischemic retina, the gene expression profile changes in response to hypoxia. [3–5]. Vascular endothelial growth factor (VEGF) is central to the pathology of retinal ischemic disease, and therapeutics that neutralize VEGF are partially effective in alleviating these pathologies [6, 7]. VEGF signaling promotes angiogenesis and vascular leakage by inducing endothelial cell proliferation, migration, and permeability. VEGF signaling contributes to severe and sight-threatening pathologies such as neovascular glaucoma, vitreous hemorrhage, and macular edema. Retinal ischemic diseases also cause and are exacerbated by chronic inflammation, with a complex interplay between inflammatory and angiogenic regulators. In retinal ischemic diseases such as diabetic retinopathy, retinal expression of proinflammatory regulators such as TNFα and ICAM-1 is increased [8, 9].

Intravitreal administration of anti-angiogenic agents targeting VEGF and photocoagulation of retinal ischemic regions are widely used for treatment of retinal ischemic disease, and are partially effective in treating these pathologies. Currently, anti-VEGF agents targeting VEGF signaling are the most commonly used therapeutics for retinal ischemic diseases, and their therapeutic effects have been reported in several studies [10–13]. However, physiological angiogenesis, which is driven in large part by VEGF, is indispensable for tissue development and survival. Anti-VEGF agents are administered intravitreally to treat ischemic retinal disease but are known to enter the bloodstream in significant amounts [14]. The side effects of decreasing of serum VEGF levels through intravitreal administration of anti-VEGF agents are currently unknown, although systemic delivery of these agents in cancer patients causes severe and potentially fatal side effects [15]. The potential side effects of anti-VEGF therapies in retinopathy of prematurity are especially controversial, as VEGF-dependent developmental processes are ongoing in premature infants [16]. On the other hand, photocoagulation also has a significant therapeutic effect in retinopathy of prematurity, although this procedure results in loss of peripheral vision [17–19]. According to our prior study using *in vivo* RVO model, photocoagulation of the ischemic region significantly decreases VEGF levels [20]. However, it could causes nonselective retinal damage including retinal inflammation [21].

To address these unmet clinical needs and theoretical gaps in knowledge, various animal models of RVO, including mice, rats, rabbits, and cats, have been developed [22]. Mice and rats are easy to house, and their retinal structures are relatively similar to humans, so they are widely used for vision models and are the most common model organisms. The rabbit RVO model employed in the present study is widely used to evaluate the potential therapeutic effects of experimental surgical procedures, as rabbits pose the additional advantage of having a larger eyeball than other rodent species [23–25]. However, the rabbit RVO model has not been extensively used for detailed analysis of the molecular mechanisms of RVO pathology due to a lack of rabbit-specific molecular tools, and because the retinal vasculature of rabbits differs from that of humans [22, 26].

In the present study, we analyzed ischemia-responsive gene expression changes in the rabbit RVO model by first identifying the temporal peak of *Vegfa* expression, which is hypoxia responsive, after induction of RVO, and subsequently performing microarray analysis of RVO and control retinas on day 7 after RVO induction, when *Vegfa* expression was highest. Our findings revealed that pro-angiogenic and inflammatory genes, which play known roles in human ischemic retinal diseases, were significantly upregulated in rabbit RVO retinas. This suggests that the rabbit RVO model is relevant for the study of ischemic retinal diseases, as the transcriptional reprogramming following RVO in rabbits recapitulated that of human ischemic retinal diseases.

## Materials and methods

### Animals

We used 2.0–3.0 kg Dutch rabbits for experiments. All experimental procedures were performed on rabbits anesthetized by intramuscular injection of ketamine hydrochloride (25 mg/kg) and xylazine hydrochloride (10 mg/kg) as previously reported [20]. Animal experiments were conducted according to the guidelines of the Association for Research in Vision and Ophthalmology Statement for the Use of Animals in Ophthalmic and Vision Research and approved by the Animal Use Committee of University of Fukui.

### RVO induction and fluorescein angiography

After rabbits were anesthetized, their pupils were dilated with tropicamide and phenylephrine (Mydrin-P; Santen Pharmaceutical, Osaka, Japan) eye drops, RVO was created by laser occlusion of the retinal veins using an argon green laser (Iris Medical Oculight Glx; IRIDEX Corporation, Mountain View, CA, USA). Induction of RVO and retinal fluorescein angiography were performed as reported in our previous study[20, 27]. The laser power was 300 mW and the duration of each ablation was 0.5 s. RVO was induced in the right eye of all animals, and the contralateral (left) eyes were used as non-RVO controls.

### Total RNA extraction and RT-qPCR

At the specified times after RVO induction, eyes were enucleated and retinas were isolated. To minimize the unexpected effects of laser treatments, the laser-irradiated region was removed using a 3-mm diameter biopsy trepan prior to sample collections. Total RNA extraction and quantitative RT-PCR were performed as previously reported [28]. Data was normalized to 18S rRNA expression. The result was not changed when we used PPIA which expressed moderately (data not shown). Statistical analysis of RT-qPCR results was performed using Microsoft Excel Office, and a two-tailed, unpaired Student’s t-test was used to assess the differences between the RVO and control groups for each time point. *P* < 0.05 was considered statistically significant.

### Microarray analysis

Microarray analysis of day 7 control and RVO retinas was conducted (n = 1). RNA integrity number (RIN) and concentration of total RNA sample were measured before performing microarray analysis by using Agilent 2100 Bioanalyzer. RIN values of control and RVO sample were 8.5 and 8.7. A Gene Chip Rabbit Gene 1.0 ST Array (Thermo Fisher Scientific) was used, and all experimental procedures were conducted according to the manufacturer’s instructions. Prior to analysis, data was normalized by RMA (**R**obust **M**ulti-array **A**verage) algorithm using R (version 3.6.0). In addition, unnamed genes and genes identified as having low signals (Max signal < 5.00 in all samples) were omitted. GO term analysis was performed using the DAVID database (https://david.ncifcrf.gov/home.jsp). Genes with >2-fold changes in expression in RVO retinas relative to control retinas were included in GO analysis. To search for human homologs, the BioMart database on the Ensembl website (http://www.ensembl.org) was used. To search for homologous genes between humans and rabbits, we referred to the official Ensembl website (http://www.ensembl.info). Gene set enrichment analysis was performed using GSEA software (https://www.gsea-msigdb.org). For gene set enrichment analysis, we used “hallmark” gene sets. Gene expression profile mapping using the R pathview package was conducted for all genes. Connectivity mapping analysis (https://portals.broadinstitute.org/cmap/) was conducted on genes with >2-fold expression change in RVO retinas relative to control retinas. The mechanism of action for each compound was referenced to the Connectivity map data library (https://clue.io/). The data are available under accession number GSE149102 at https://www.ncbi.nlm.nih.gov/geo/query/acc.cgi?acc=GSE149102.

## Results

### Maximal *Vegf* expression 7 days after RVO induction

**Fig 1:**
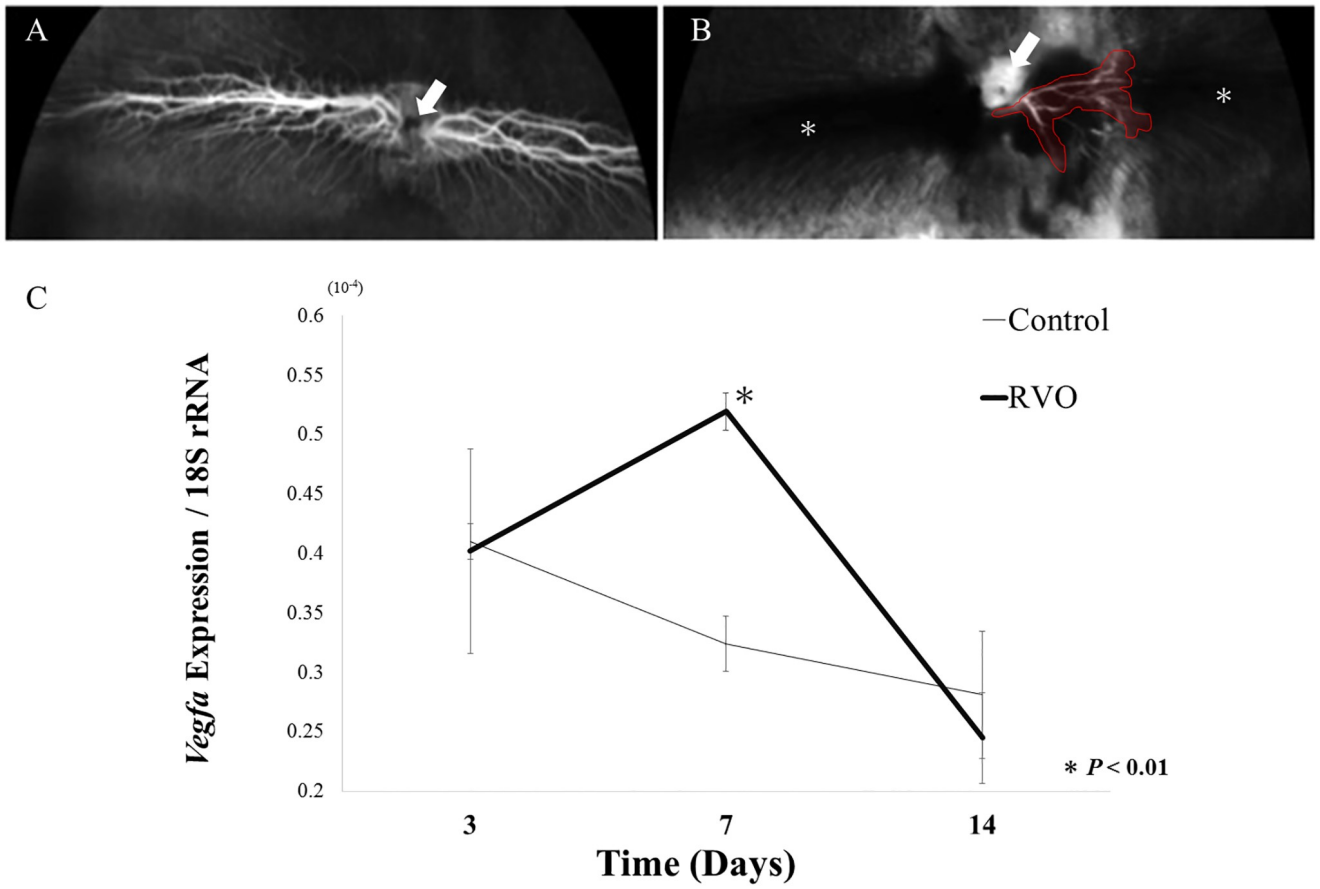
Fluorescein angiography *Vegfa* RT-qPCR. **(A)** Fluorescein angiography (FA) revealed that control retinas exhibited normal blood flow. Arrows indicates optic nerve head. **(B)** In ischemic retinas 7 days after RVO induction, blood flow was predominately blocked. Some revascularized vessels (surrounded with *red*) were observed, but the vessels did not reach the periphery area (*). **(C)** Retinal *Vegfa* expression on days 3, 7 and 14 after RVO induction. *Vegfa* expression was significantly increased in RVO retinas compared to control only on day 7 after RVO induction. RT-qPCR results were normalized to 18S rRNA expression. Data are expressed as mean ± SE (n = 3). (* *P* < 0.01, day 7 RVO versus day 7 control retina.).

In prior studies of the ischemic response after RVO induction, we identified that retinal VEGF protein levels are maximal at day 7 post-RVO [20]. However, we had not previously examined the timing of the transcriptional response to RVO-induced ischemia. Thus, to determine the optimal time point for microarray analysis, we performed fluorescein angiography (FA) and RT-qPCR measurement of *Vegfa* expression to evaluate RVO severity and the ischemic transcriptional response, respectively. FA images captured 7 days after induction of RVO demonstrated that blood flow was blocked near the optic disc in RVO retinas compared with controls (Fig 1A and 1B). In the RVO retinas, a partial reperfusion region was present, but this region did not reach the peripheral area (Fig 1B). These findings suggested that the ischemic state of the retina was sufficiently maintained for at least 7 days after RVO induction. Next, to assess ischemia-induced transcriptional changes over time, we quantified *Vegfa* expression by RT-qPCR (Fig 1C). At 3 days and 14 days after RVO induction, *Vegfa* expression was not significantly different between RVO and control retinas. Contrastingly, retinal *Vegfa* was significantly increased at day 7 post-RVO (Fig 1C). Collectively, these findings suggested that in the rabbit RVO model, ischemic pathology was most severe 7 days after induction of RVO. Therefore, this time point was used for microarray analysis.

### Upregulation of angiogenic and inflammatory mediators in RVO retinas

In the rabbit RVO model, the ischemic transcriptional response was induced 7 days after RVO induction, rather than immediately after induction of ischemia with RVO. To investigate the detailed molecular mechanism of the transcriptional response to ischemia, we performed a microarray analysis of day 7 samples, which exhibited the greatest upregulation of *Vegfa* expression, suggesting that the ischemic transcriptional response was most active at this time point. Genes that exhibited significant expression changes were extracted for analysis. In a total of 13,563 genes evaluated, 520 exhibited a greater than 2-fold expression change. These genes included 456 upregulated genes and 64 downregulated genes (S1 Table). Expression of the pro-angiogenic factors *Dcn* and *Mmp1* and pro-inflammatory factors *Mmp12* and *Cxcl13* were markedly increased in RVO retinas [29, 30].

The regulatory role of decorin (DCN) in angiogenesis has been demonstrated in several studies using mice and human cells [31–33]. However, whether DCN functions to promote or suppress angiogenesis is tissue type- and context-dependent [34]. Although the function of DCN in the rabbit RVO model cannot be deduced in the present analysis, expression of *Mmp1*, a known DCN target gene, was increased. Furthermore, expression of *Rest*, which contributes to ischemic neuronal insults, was significantly increased [35]. In the context of ischemia-induced neuronal death, *Rest* is upregulated in response to ischemia, and forms a REST/Cofactor for REST (CoREST) complex, which contains a histone deacetylase complex (HDAC) that mediates epigenetic gene silencing [36]. Further, among REST target genes, the expression of *Nefh* (0.23-fold) and *Gria2* (0.65-fold) were significantly downregulated in the RVO group. Furthermore, the expression of genes of as yet unknown function, such as *LOC100341104* and *LOC100008687*, were increased. Although potential human homologs for these genes have not yet been identified, they could play important roles as unknown ischemic response genes. Contrastingly, *Nrn1*, *SLlc7a6*, and *Nefh* were dramatically downregulated in RVO retinas. Further studies are needed to elucidate the potential contribution of this interaction to the pathology of RVO.

Subsequently, functional clustering of genes with 2-fold or greater upregulation was conducted using the Database for Annotation, Visualization and Integrated discovery (DAVID, https://david.ncifcrf.gov/home.jsp) (Table 1). Most strikingly, inflammatory mediators, such as *Tlr3*, *Tlr4*, and *Ripk2*, were upregulated (Table 1). Additionally, PI3K-Akt signaling, which is associated with angiogenesis, was identified as a potentially significant pathway. Expression of Oncostatin M receptor (*Osmr*), which is related to PI3K-AKT signaling pathway (KEGG_PATHWAY; ocu04151), was markedly increased in RVO retinas. OSMR was reported to be involved in activation of the JAK-STAT and MAPK signaling pathways [37]. An *in vivo* study using the rat choroidal neovascularization (CNV) model reported that administration of PI3K inhibitors not only decreases CNV lesion size and vascular leakage, but also significantly decreases VEGF expression [38].

**Table 1:** DAVID analysis results.

Category	Term	p-value	Fold Enrichment
**Annotation Cluster 1**	**Enrichment Score: 5.47**		
UP_KEYWORDS	Immunity	2.15E-14	12.5
GOTERM_BP_DIRECT	GO:0045087~innate immune response	1.99E-07	5.39
UP_KEYWORDS	Innate immunity	2.75E-07	12.8
UP_KEYWORDS	Inflammatory response	4.27E-06	11.3
GOTERM_MF_DIRECT	GO:0004888~transmembrane signaling receptor activity	1.74E-03	6.72
GOTERM_BP_DIRECT	GO:0050707~regulation of cytokine secretion	6.84E-03	9.77
GOTERM_BP_DIRECT	GO:0002755~MyD88-dependent toll-like receptor signaling pathway	8.03E-02	6.28
**Annotation Cluster 2**	**Enrichment Score: 2.59**		
KEGG_PATHWAY	ocu04510: Focal adhesion	2.57E-04	2.87
KEGG_PATHWAY	ocu04512: ECM-receptor interaction	8.29E-04	3.98
KEGG_PATHWAY	ocu04151: PI3K-Akt signaling pathway	7.72E-02	1.62
**Annotation Cluster 3**	**Enrichment Score: 2.34**		
GOTERM_BP_DIRECT	GO:0071223~cellular response to lipoteichoic acid	7.24E-04	19.5
GOTERM_BP_DIRECT	GO:0032755~positive regulation of interleukin-6 production	1.93E-03	6.51
GOTERM_BP_DIRECT	GO:0032760~positive regulation of tumor necrosis factor production	6.84E-02	4.19
**Annotation Cluster 4**	**Enrichment Score: 1.39**		
GOTERM_BP_DIRECT	GO:0032755~positive regulation of interleukin-6 production	1.93.E-03	6.51
GOTERM_BP_DIRECT	GO:0046330~positive regulation of JNK cascade	4.97.E-02	3.57
GOTERM_BP_DIRECT	GO:0032722~positive regulation of chemokine production	5.18.E-02	7.99
GOTERM_BP_DIRECT	GO:0051092~positive regulation of NF-kB transcription factor activity	5.07.E-01	1.48

Genes increased 2-fold or greater (456 genes) were included in analysis. The remarkable clusters and their enrichment scores are listed.

### Gene set enrichment analysis of human homologs

**Fig 2:**
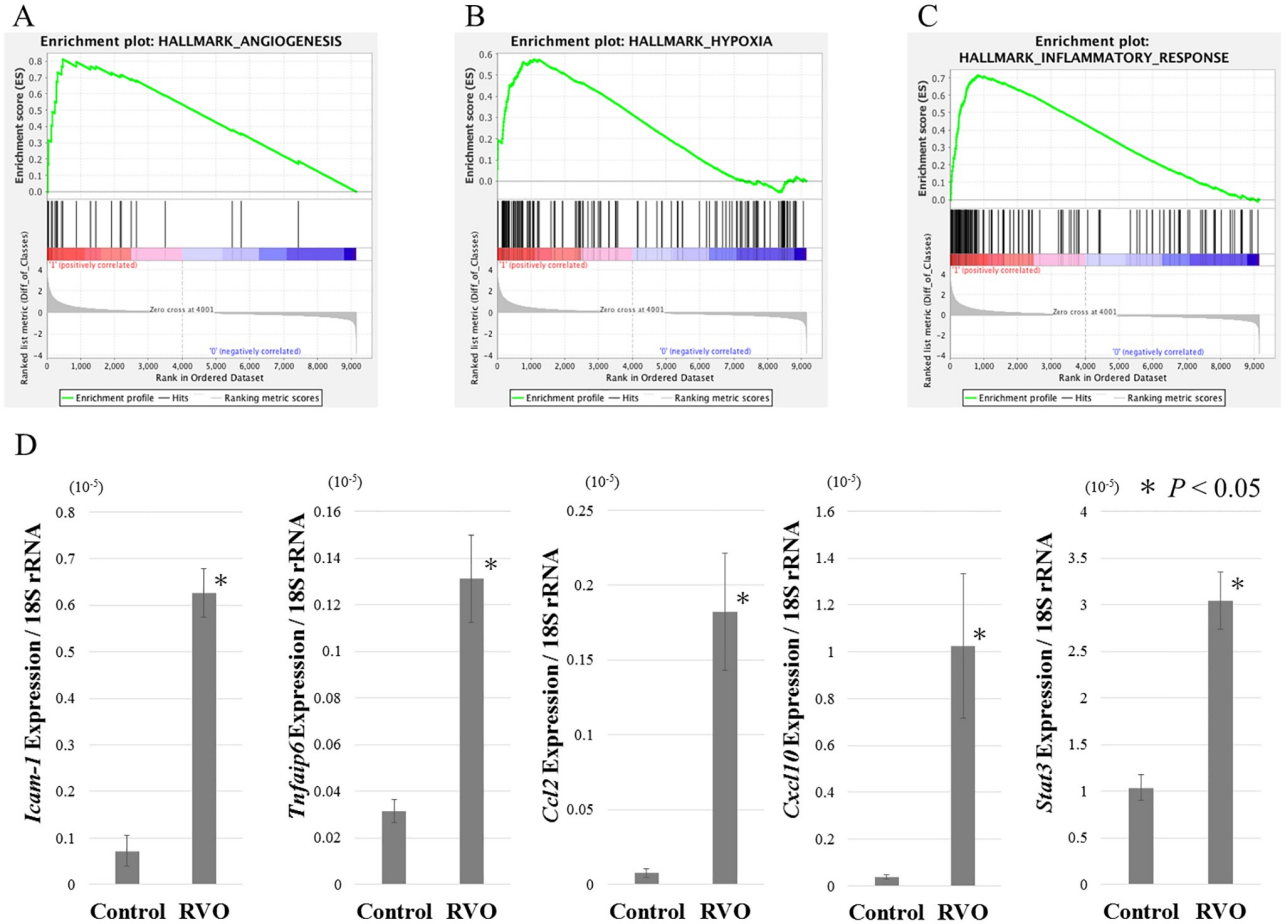
Enrichment plots of remarkable gene sets. Hypoxia, angiogenesis, and inflammatory pathways were significantly upregulated in RVO retinas. Plots are shown for **(A)** angiogenesis, **(B)** hypoxia, and **(C)** inflammatory response gene sets. **(D)** RT-qPCR result on days 7 after RVO induction. *Tnfaip6*, *Icam-1*, *Ccl2*, *Cxcl10*, *Stat3* expressions were significantly increased in RVO retinas compared to control retina. RT-qPCR results were normalized to 18S rRNA expression. Data are expressed as mean ± SE (n = 3). (* *P* < 0.05, RVO versus control retina.).

Microarray analysis of rabbit gene expression profiles in RVO revealed that expression of pro-inflammatory and angiogenic factors was significantly increased in the rabbit RVO model. Next, to evaluate the similarity of the rabbit RVO model to human ischemic retinal disease, we performed an analysis targeting only genes in which homology with human genes was confirmed. In this analysis, only 9,436 genes were identified as human homologs in the Ensembl-BioMart database (http://www.ensembl.org), so were used in analysis [39, 40]. Enrichment analysis using Gene Set Enrichment Analysis (GSEA, https://www.gsea-msigdb.org) was performed to extract the human gene sets that were induced or suppressed by retinal ischemia [41]. We uploaded the expression data of human homologs in the control and RVO groups, and the gene sets significantly upregulated in the RVO group were extracted (Table 2). Gene sets significantly affected by induction of retinal ischemia were related to hypoxia, angiogenesis, and inflammation. Among the inflammatory pathways enriched in our analysis, the IL6, JAK-STAT [42, 43], TNFα, and NF-κB pathways [44, 45] exacerbate the pathology of retinal disease. However, the expression levels of genes included in each upregulated gene set were not increased equally. Thus, we extracted the genes contained in each gene set, and ranked the genes by relative expression change (S2 Table). In addition, enrichment plots [41] for the above-mentioned gene sets were generated to evaluate the expression tendencies of the genes included in each group (Fig 2A–2C). The middle panel of each plot shows the ranking of each gene. Genes upregulated in the RVO group relative to the control group are displayed on the left side. The height of the enrichment profile displayed at the top indicates the enrichment score for each gene set, and when many highly ranked genes are included, the peak will be higher on the left side. The lower panel shows the ranking of all input genes and the relative levels of expression change. In the angiogenesis gene set, *Timp1*, *Lum*, and *Lpl* were significantly upregulated (Fig 2A). However, expression of *Stc* and *Pglyrp1* was unchanged in RVO retinas. In the hypoxia gene set, expression of *Dcn*, *Adm*, *Jun*, and *Fos* were markedly upregulated, while *Tnfaip3* was downregulated (Fig 2B). In the inflammatory response gene set, top ranked genes with the greatest upregulation included *Cd14*, *Ccl2*, and *Rgs1* (Fig 2C). Clear peaks appeared at the top of the ranking for gene sets associated with angiogenesis, hypoxia, and the inflammatory response, indicating that these genes were significantly upregulated in RVO retinas. In addition, to confirm our microarray result, we performed RT-qPCR for *Icam-1*, *Tnfaip6*, *Ccl2*, *Cxcl10*, *Stat3* genes (Fig 2D). These genes were related to TNF or STAT pathway and significantly upregulated both in microarray and RT-qPCR. This result emphasized the possibility that our analysis was correct and that TNFα and STAT pathways are important for the pathology of RVO.

**Table 2:** Gene set enrichment analysis results.

MSigDB Name (HALLMARK)	NES	p-value	FDR
IL6_JAK_STAT3_SIGNALING	2.42	0.00.E+00	0.00.E+00
MYOGENESIS	2.34	0.00.E+00	0.00.E+00
EPITHELIAL_MESENCHYMAL_TRANSITION	2.34	0.00.E+00	0.00.E+00
INTERFERON_GAMMA_RESPONSE	2.30	0.00.E+00	0.00.E+00
COAGULATION	2.29	0.00.E+00	0.00.E+00
INTERFERON_ALPHA_RESPONSE	2.28	0.00.E+00	0.00.E+00
INFLAMMATORY_RESPONSE	2.27	0.00.E+00	0.00.E+00
ALLOGRAFT_REJECTION	2.25	0.00.E+00	0.00.E+00
COMPLEMENT	2.15	0.00.E+00	0.00.E+00
TNFA_SIGNALING_VIA_NFKB	2.10	0.00.E+00	0.00.E+00
ANGIOGENESIS	2.04	0.00.E+00	0.00.E+00
APOPTOSIS	2.04	0.00.E+00	0.00.E+00
IL2_STAT5_SIGNALING	2.02	0.00.E+00	0.00.E+00
KRAS_SIGNALING_UP	1.86	0.00.E+00	4.13.E-04
HYPOXIA	1.85	0.00.E+00	3.86.E-04
MITOTIC_SPINDLE	1.85	0.00.E+00	3.62.E-04
APICAL_JUNCTION	1.84	0.00.E+00	4.11.E-04
TGF_BETA_SIGNALING	1.73	1.37.E-03	3.10.E-03
CHOLESTEROL_HOMEOSTASIS	1.65	8.21.E-03	8.11.E-03
G2M_CHECKPOINT	1.65	0.00.E+00	7.99.E-03
UV_RESPONSE_DN	1.64	3.52.E-03	8.76.E-03
GLYCOLYSIS	1.63	1.12.E-03	8.95.E-03
P53_PATHWAY	1.61	1.17.E-03	1.09.E-02
E2F_TARGETS	1.56	6.90.E-03	1.96.E-02
ESTROGEN_RESPONSE_LATE	1.50	1.97.E-02	3.55.E-02
XENOBIOTIC_METABOLISM	1.49	1.06.E-02	3.94.E-02

Human homologs (9,436 genes) were included in analysis. Significantly upregulated gene sets are listed. Normalized enrichment score (NES), p-value, and false discovery rate (FDR) are specified for each gene set. *P* < 0.05 was considered statistically significant.

These results suggested that significant differences in gene expression profiles were observed in rabbit RVO retinas that were similar to those of human ischemic retinal diseases. This supports the relevance of the rabbit RVO model to human retinal ischemic disease.

### Visualization of gene expression profiles

**Fig 3:**
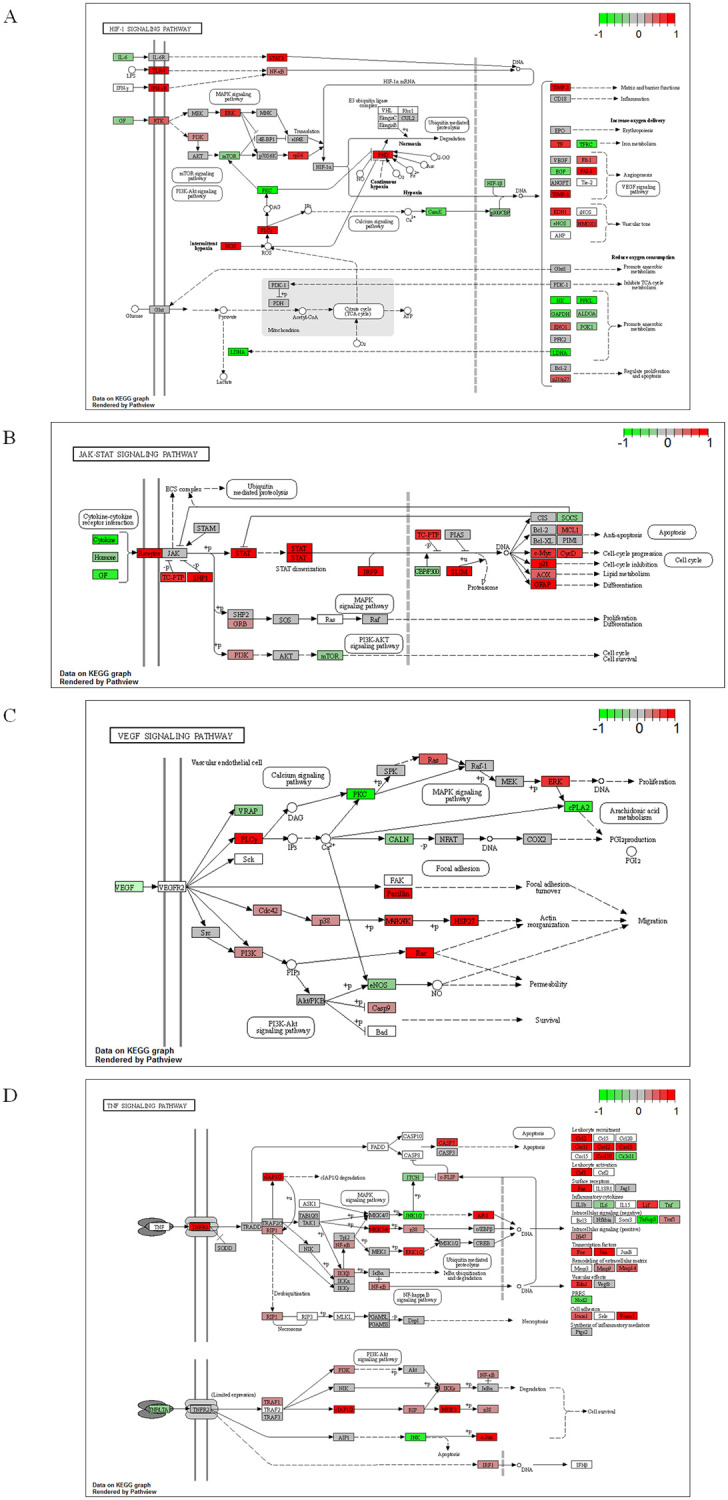
Visualization of gene expression profiles in KEGG pathway mapping. Each cell represents the log2 ratio gene expression data of the RVO group normalized to the control group. The coloration indicates upregulated (Red) and downregulated (Green) genes. Pathway maps of **(A)** HIF-1α, **(B)** VEGF, **(C)** TNFα, and **(D)** JAK-STAT signaling pathways. The analysis was performed using the R pathview package.

GESA analysis identified pathways related to pathologies such as chronic inflammation, increased vascular permeability, and pathological angiogenesis, which are common features of retinal ischemic disease. However, not all genes involved in each pathway were equivalently upregulated at the transcriptional level, as demonstrated by the enrichment plots (Fig 2). Therefore, we next visualized the expression changes of the gene sets that comprise the hypoxic response, angiogenesis, and pro-inflammatory pathways (Fig 3). This approach allows detailed analysis of downstream signaling altered by activation of each pathway. The gene expression changes in the RVO samples are indicated by color on the KEGG pathway map, generated using the R pathview package (Fig 3A–3D) [46]. First, we visualized the HIF-1α and VEGF pathways, which are widely recognized as ischemic responsive genes in the rabbit RVO model. Analysis of the HIF-1α pathway revealed transcriptional changes in genes upstream and downstream of HIF-1α (Fig 3A). Expression analysis of downstream factors, which directly regulated by HIF-1α, revealed that expression of angiogenic mediators such as *Timp1* and *Pai-1* were upregulated, but that expression of inflammatory factors were unchanged. VEGF pathway mapping revealed that MAPK signaling, which regulates endothelial cell proliferation, was activated in RVO retinas (Fig 3B). Further, expression of Paxillin (*Pxn*), which is associated with focal adhesion, was upregulated. PXN is downstream of VEGF signaling, and was also extracted in DAVID analysis (Table 1). Next, we visualized the expression profile of the JAK-STAT, TNFα, and NF-κB pathways, which were upregulated in the enrichment analysis (Fig 3C and 3D). Expression of downstream factors of each pathway was changed by RVO, and was suggestive of pathway activation. These data suggested that the JAK-STAT, TNFα, and NF-κB pathways are likely to contribute to the pathology of rabbit RVO, and potentially to human retinal ischemic diseases. Analysis of JAK-STAT signaling revealed a marked increase not only in expression of the *Stat* gene itself, but also of *Shp1(Ptpn6)*, which suppress the activity of this pathway in a negative feedback loop (Fig 3C). However, expression of cell cycle-related factors downstream of STAT, such as the *c-Myc* and *p21* genes, was upregulated by RVO. This seemingly contradictory result suggested that the balance of JAK/STAT signaling activation and inhibition could be important to the pathology of RVO. On the other hand, chemokines, such as *Ccl1* and *Cxcl1*, *2*, *3*, and *10* were significantly upregulated in RVO (Fig 3D). These genes are pro-inflammatory factors downstream of TNFα and NF-κB signaling, and could potentially be effective therapeutic targets for inflammation secondary to retinal ischemia.

The pathview analysis findings suggested that the downstream factors of the four different pathways extracted by GSEA, including HIF-1α, JAK-STAT, TNFα, and VEGF, exhibited gene expression changes consistent with pathway activation.

### Connectivity map analysis

Pathview analysis of human homologs revealed that the changes in gene expression profile in the rabbit RVO model were consistent with the pathology of human retinal ischemic disease. Therefore, we searched for chemical compounds that could potentially be used as novel therapeutic agents based on the gene expression profiles of the rabbit RVO model. We used a connectivity map database (https://portals.broadinstitute.org/cmap/), which allowed us to search for compounds that induce similar gene expression profile changes in human cancer cells based on the uploaded gene names [47]. Among the human homologs (9,436 genes), we analyzed 387 genes with a greater than 2-fold difference between RVO and control samples (upregulated: 333 genes, downregulated: 54 genes). We focused on compounds that induced gene expression profile changes inverse to those detected in our microarray analysis of RVO retinas, and extracted the top 20 compounds (Table 3). Although the effects of these genes in RVO have not yet been clarified, DNA synthesis inhibitors, melanin inhibitors, and adenosine receptor antagonists were extracted. Remarkably, the identified compounds included MG-262 [48, 49] and parthenolide [50], inhibitors of the NF-κB pathway.

**Table 3:** Connectivity mapping analysis results.

Name	Enrichment	p-value	Mechanism of action
15-delta prostaglandin J2	-0.591	0.00.E+00	-
MG-262	-0.965	1.00.E-04	-
5182598	-0.991	2.00.E-04	-
parthenolide	-0.892	3.00.E-04	NFκB pathway inhibitor
foliosidine	-0.723	9.00.E-04	-
6-bromoindirubin-3'-oxime	-0.683	9.00.E-04	-
lomustine	-0.852	9.00.E-04	DNA synthesis inhibitor
ambroxol	-0.825	1.80.E-03	sodium channel blocker
PHA-00816795	-0.964	2.90.E-03	-
5194442	-0.799	3.20.E-03	-
diloxanide	-0.769	5.70.E-03	protein synthesis inhibitor
pipemidic acid	-0.830	9.80.E-03	-
thiamine	-0.822	1.13.E-02	vitamin B
spaglumic acid	-0.925	1.17.E-02	-
monobenzone	-0.701	1.66.E-02	melanin inhibitor
tubocurarine chloride	-0.698	1.76.E-02	-
aminophylline	-0.687	2.06.E-02	adenosine receptor antagonist
PNU-0251126	-0.575	2.07.E-02	-
aceclofenac	-0.678	2.38.E-02	prostanoid receptor antagonist
ticarcillin	-0.773	2.41.E-02	lactamase inhibitor

Connectivity mapping analysis of genes with greater than 2-fold expression change between control and RVO retinas (upregulated: 333 genes, downregulated: 54 genes). The top 20 chemical compounds that induce transcriptional reprogramming inverse to the input data are listed.

The GSEA and pathview analyses suggested that the NF-κB pathway was significantly upregulated in RVO retinas. Connectivity mapping analysis supported the importance of the NF-κB pathway in RVO.

## Discussion

Various animal models using mice, rats, rabbits, and cats have been developed as experimental systems that mimic retinal ischemic diseases. The rabbit RVO model used in the present study has the advantage of having a large eyeball, and therefore relative ease of performing surgical procedures. Therefore, rabbit RVO is commonly used to evaluate the efficacy and outcomes of experimental surgical interventions [23–25]. However, the retinal vasculature of rabbits is not distributed entire retina unlike humans (Fig 1A). Because of this anatomical difference, it was controversial that the course of the disease or its response to a treatment may not be the same as in humans [22, 26]. The present study is the first to use bioinformatics approaches to elucidate the molecular pathology of the rabbit RVO model. Further, we conducted an analysis focusing on comparison with other species, especially humans.

Retinal ischemia occurs secondary to various aberrations such as diabetes, hypertension, and preterm birth, and causes pathological angiogenesis and chronic inflammation [1, 2]. According to the present analysis, the expression levels of angiogenic regulators and inflammatory mediators were significantly upregulated in the rabbit RVO model (Table 1). Previous reports using clinical samples from RVO patients identified that these angiogenic and inflammatory mediators are upregulated in the context of human RVO [51, 52]. Additionally, genes associated with induction of the hypoxic response, angiogenesis, and inflammation were activated, as indicated by the analysis of human homolog genes differentially expressed in rabbit RVO (Table 2). These findings demonstrated that the rabbit RVO model, in which ischemia is artificially induced by laser photocoagulation of retinal veins, exhibited similar transcriptional reprogramming to that observed in human disease, supporting the relevance of rabbit RVO to human ischemic retinal disease.

A prior gene expression profiling study by Martin et al. in the mouse RVO model reported that genes related to the induction of angiogenesis and inflammation are upregulated in RVO groups compared with sham treated groups [53]. The GSEA conducted in the present study confirmed that signaling pathways related to in IL6, TNFα, and TGFβ were activated in rabbit RVO retinas (Table 2). Additionally, *Ccl2* was upregulated (S1 Table). However, in the mouse model, Martin et al. suggested that the expression of *Il6*, *Ccl2*, *Tnf* were both increased in RVO and sham treated groups compared with untreated group. They concluded that the gene expression profile in a mouse RVO model was related to laser-induced retinal damage. On the other hand, rabbit model showed hypoxia-dependent gene expression changes in RVO sample, but not in control group. These result indicated that the rabbit model is more appropriate as an evaluation system for the pathogenesis of RVO than other animal models.

In the ischemic retina, nutrients and oxygen are deficient, resulting in irreversible damage to retinal neurons. REST corepressor complex-dependent regulation of gene expression contributes to ischemia-induced neural insults [35]. This complex binds to the Neuron Restrictive Silencer Element (NRSE)/ repressor element 1(RE1) site present in the promoter regions of target genes, resulting in silencing of gene expression. Our analysis identified that *Rest* was upregulated in rabbit RVO, and that *Nfeh* and *Gria2*, target genes of the REST corepressor complex, were downregulated in rabbit RVO (S1 Table). Furthermore, the homology of these genes between rabbit and human has been confirmed. Taken together, these findings suggested that retinal ischemia could cause epigenetic modifications. A prior study using primary retinal ganglion cells (RGCs) derived from rats reported that sequestration of REST by a plasmid containing the NRSE/RE1 element enhances RGC neurite outgrowth [54]. However, the relationship between REST and retinal ischemia, especially in relation to epigenetic modifications, remains incompletely understood. The findings of the present study provide insights into the molecular pathology of rabbit RVO, and its similarity to other RVO models and human ischemic retinal disease.

VEGF drives the pathology of many retinal ischemic diseases. Retinal ischemia induces VEGF expression, driving pathological angiogenesis and increasing vascular permeability [6, 7]. In the present study, we determined that 7 days after induction of retinal ischemia with RVO, *Vegfa* mRNA levels were maximal, suggesting that the ischemic response was maximal at this time point. We therefore used day 7 RVO samples for microarray analysis (Fig 1C).

Pathview analysis revealed that expression levels of VEGF target genes were upregulated in rabbit RVO (Fig 3B). Among pathways downstream of VEGF, genes involved in the PI3K-Akt pathway, which regulates vascular permeability, were significantly upregulated (Table 1). In addition to the microarray analysis findings in the present study, our prior study demonstrated that photocoagulation of the ischemic region in the rabbit RVO model significantly decreased retinal VEGF levels [20]. These findings underscore the similarity of the rabbit RVO model to human disease, suggesting that this ischemic model could be used for the study of angiogenic regulators.

In addition, genes related to migration of vascular endothelial cells, such as the PI3K pathway, including *Osmr* (Table 1), *Pxn* (Fig 3B), and *Mmp* genes (S1 Table), were upregulated in rabbit RVO retinas. MMP12 belongs to the superfamily of macrophage-secreted matrix metalloproteinases (MMPs), and is related to induction of retinal angiogenesis [55]. Vascular endothelial cell migration is an essential process of angiogenesis, and induction of these genes in the rabbit RVO model is a notable finding because it emphasizes the validity of this model as an ischemic model. Interestingly, prior studies have reported that the promoter region of *Pxn* contains binding sequences for the transcription factors NF-κB, STAT3, and AP-1 [56, 57]. Expression of these transcription factors was markedly increased in the rabbit RVO model (Fig 3). Although microarray analysis is only capable of measuring transcript levels, our findings were consistent with prior studies of other related models, supporting the relevance of our experimental system.

In addition to angiogenic factors, inflammatory mediators were also upregulated in rabbit RVO retinas (Tables 1 and 2). However, because RVO is induced by photocoagulation of Rose Bengal, the off-target effects of laser irradiation independent of ischemia on the retina must be carefully considered. Importantly, a prior study of a mouse RVO model demonstrated that laser damage induces a robust inflammatory response independent of retinal ischemia [53]. On the other hand, proteomic studies of a porcine RVO model failed to demonstrate that laser damage alone activated an inflammatory response in this context, suggesting that the effects of laser damage on inflammation are species- and model-dependent [58]. However, these reports concluded that the ratio of the laser irradiation site to the hypoxic area is important for laser damage-induced inflammation, and that the use of larger animals such as rats, pigs, and rabbits could reduce the artifacts generated by laser irradiation independent of ischemia. Furthermore, when we collected samples for analysis, the laser-irradiated area was excluded. Therefore, the direct effects of laser damage can be excluded from the results of the present study. Even considering the potential unexpected indirect effects of laser irradiation, our results suggested that retinal ischemia induced the inflammatory response independent of laser irradiation damage.

The present study is the first to demonstrate that transcription of angiogenic and pro-inflammatory mediators was increased in the rabbit RVO model. Microarray analysis targeting homologs and subsequent pathway analysis is an effective method to compare the pathology of experimental disease models with human disease. The results of these analyses strongly suggested that the rabbit RVO model is a valid model of retinal ischemic disease, and poses the additional advantage of relative ease of surgical manipulation. We expect that novel approaches to treatment of retinal ischemic disease could be developed using the rabbit RVO model.

## Supporting information

S1 Table List of significantly altered genes. (XLSX)

S2 Table Expression change rankings for each gene set. (XLSX)

## References

[1] Duh EJ, Sun JK, Stitt AW. Diabetic retinopathy: current understanding, mechanisms, and treatment strategies. JCI Insight. 2017;2(14). Epub 2017/07/21. 10.1172/jci.insight.93751 . 28724805 PMC5518557

[2] Shah PK, Prabhu V, Karandikar SS, Ranjan R, Narendran V, Kalpana N. Retinopathy of prematurity: Past, present and future. World J Clin Pediatr. 2016;5(1):35–46. Epub 2016/02/11. 10.5409/wjcp.v5.i1.35 . 26862500 PMC4737691

[3] Dioum EM, Clarke SL, Ding K, Repa JJ, Garcia JA. HIF-2alpha-haploinsufficient mice have blunted retinal neovascularization due to impaired expression of a proangiogenic gene battery. Invest Ophthalmol Vis Sci. 2008;49(6):2714–20. Epub 2008/02/19. 10.1167/iovs.07-1469 . 18281611

[4] Jiang J, Xia XB, Xu HZ, Xiong Y, Song WT, Xiong SQ, et al Inhibition of retinal neovascularization by gene transfer of small interfering RNA targeting HIF-1alpha and VEGF. J Cell Physiol. 2009;218(1):66–74. Epub 2008/09/04. 10.1002/jcp.21566 . 18767037

[5] Majmundar AJ, Wong WJ, Simon MC. Hypoxia-inducible factors and the response to hypoxic stress. Mol Cell. 2010;40(2):294–309. Epub 2010/10/23. 10.1016/j.molcel.2010.09.022 . 20965423 PMC3143508

[6] Boulton M, Foreman D, Williams G, McLeod D. VEGF localisation in diabetic retinopathy. Br J Ophthalmol. 1998;82(5):561–8. Epub 1998/08/26. 10.1136/bjo.82.5.561 . 9713066 PMC1722605

[7] Bhisitkul RB. Vascular endothelial growth factor biology: clinical implications for ocular treatments. Br J Ophthalmol. 2006;90(12):1542–7. Epub 2006/11/23. 10.1136/bjo.2006.098426 . 17114590 PMC1857529

[8] Miyamoto K, Khosrof S, Bursell SE, Rohan R, Murata T, Clermont AC, et al Prevention of leukostasis and vascular leakage in streptozotocin-induced diabetic retinopathy via intercellular adhesion molecule-1 inhibition. Proc Natl Acad Sci U S A. 1999;96(19):10836–41. Epub 1999/09/15. 10.1073/pnas.96.19.10836 . 10485912 PMC17969

[9] Wang J, Xu X, Elliott MH, Zhu M, Le YZ. Muller cell-derived VEGF is essential for diabetes-induced retinal inflammation and vascular leakage. Diabetes. 2010;59(9):2297–305. Epub 2010/06/10. 10.2337/db09-1420 . 20530741 PMC2927953

[10] Kreutzer TC, Alge CS, Wolf AH, Kook D, Burger J, Strauss R, et al Intravitreal bevacizumab for the treatment of macular oedema secondary to branch retinal vein occlusion. Br J Ophthalmol. 2008;92(3):351–5. Epub 2008/01/24. 10.1136/bjo.2007.123513 . 18211925

[11] Kriechbaum K, Michels S, Prager F, Georgopoulos M, Funk M, Geitzenauer W, et al Intravitreal Avastin for macular oedema secondary to retinal vein occlusion: a prospective study. Br J Ophthalmol. 2008;92(4):518–22. Epub 2008/01/24. 10.1136/bjo.2007.127282 . 18211942

[12] Higashiyama T, Sawada O, Kakinoki M, Sawada T, Kawamura H, Ohji M. Prospective comparisons of intravitreal injections of triamcinolone acetonide and bevacizumab for macular oedema due to branch retinal vein occlusion. Acta Ophthalmol. 2013;91(4):318–24. Epub 2011/12/03. 10.1111/j.1755-3768.2011.02298.x . 22132711

[13] Stahl A, Lepore D, Fielder A, Fleck B, Reynolds JD, Chiang MF, et al Ranibizumab versus laser therapy for the treatment of very low birthweight infants with retinopathy of prematurity (RAINBOW): an open-label randomised controlled trial. Lancet. 2019;394(10208):1551–9. Epub 2019/09/17. 10.1016/S0140-6736(19)31344-3 . 31522845 PMC12316478

[14] Avery RL, Castellarin AA, Steinle NC, Dhoot DS, Pieramici DJ, See R, et al SYSTEMIC PHARMACOKINETICS AND PHARMACODYNAMICS OF INTRAVITREAL AFLIBERCEPT, BEVACIZUMAB, AND RANIBIZUMAB. Retina. 2017;37(10):1847–58. Epub 2017/01/21. 10.1097/IAE.0000000000001493 . 28106709 PMC5642319

[15] Saif MW, Mehra R. Incidence and management of bevacizumab-related toxicities in colorectal cancer. Expert Opin Drug Saf. 2006;5(4):553–66. Epub 2006/06/16. 10.1517/14740338.5.4.553 . 16774493

[16] Wu WC, Lien R, Liao PJ, Wang NK, Chen YP, Chao AN, et al Serum levels of vascular endothelial growth factor and related factors after intravitreous bevacizumab injection for retinopathy of prematurity. JAMA Ophthalmol. 2015;133(4):391–7. Epub 2015/01/09. 10.1001/jamaophthalmol.2014.5373 . 25569026

[17] Spranger J, Hammes HP, Preissner KT, Schatz H, Pfeiffer AF. Release of the angiogenesis inhibitor angiostatin in patients with proliferative diabetic retinopathy: association with retinal photocoagulation. Diabetologia. 2000;43(11):1404–7. Epub 2000/12/29. 10.1007/s001250051546 . 11126410

[18] Stefansson E. The therapeutic effects of retinal laser treatment and vitrectomy. A theory based on oxygen and vascular physiology. Acta Ophthalmol Scand. 2001;79(5):435–40. Epub 2001/10/12. 10.1034/j.1600-0420.2001.790502.x . 11594975

[19] Spaide RF. Prospective study of peripheral panretinal photocoagulation of areas of nonperfusion in central retinal vein occlusion. Retina. 2013;33(1):56–62. Epub 2012/12/28. 10.1097/IAE.0b013e3182641875 . 23269405

[20] Gozawa M, Takamura Y, Miyake S, Matsumura T, Morioka M, Yamada Y, et al Photocoagulation of the Retinal Nonperfusion Area Prevents the Expression of the Vascular Endothelial Growth Factor in an Animal Model. Invest Ophthalmol Vis Sci. 2017;58(13):5946–53. Epub 2017/11/04. 10.1167/iovs.17-22739 . 29098298

[21] Cui JZ, Wang XF, Hsu L, Matsubara JA. Inflammation induced by photocoagulation laser is minimized by copper chelators. Lasers Med Sci. 2009;24(4):653–7. Epub 2008/06/21. 10.1007/s10103-008-0577-8 . 18566852 PMC3947381

[22] Khayat M, Lois N, Williams M, Stitt AW. Animal Models of Retinal Vein Occlusion. Invest Ophthalmol Vis Sci. 2017;58(14):6175–92. Epub 2017/12/10. 10.1167/iovs.17-22788 . 29222552

[23] Larsson J, Carlson J, Olsson SB. Ultrasound enhanced thrombolysis in experimental retinal vein occlusion in the rabbit. Br J Ophthalmol. 1998;82(12):1438–40. Epub 1999/02/04. 10.1136/bjo.82.12.1438 . 9930279 PMC1722462

[24] Rowley SA, Vijayasekaran S, Yu PK, McAllister IL, Yu DY. Retinal toxicity of intravitreal tenecteplase in the rabbit. Br J Ophthalmol. 2004;88(4):573–8. Epub 2004/03/20. 10.1136/bjo.2003.027466 . 15031179 PMC1772065

[25] Nguyen VP, Li Y, Zhang W, Wang X, Paulus YM. High-resolution multimodal photoacoustic microscopy and optical coherence tomography image-guided laser induced branch retinal vein occlusion in living rabbits. Sci Rep. 2019;9(1):10560 Epub 2019/07/25. 10.1038/s41598-019-47062-2 . 31332266 PMC6646378

[26] Ameri H, Ratanapakorn T, Rao NA, Chader GJ, Humayun MS. Natural course of experimental retinal vein occlusion in rabbit; arterial occlusion following venous photothrombosis. Graefes Arch Clin Exp Ophthalmol. 2008;246(10):1429–39. Epub 2008/07/22. 10.1007/s00417-008-0878-4 . 18642023

[27] Kakimoto H, Takamura Y, Arimura S, Miyake S, Matsumura T, Gozawa M, et al Effect of 0.05% Difluprednate Ophthalmic Emulsion on Proinflammatory Cytokine Levels After Retinal Laser Photocoagulation in Rabbits. J Ocul Pharmacol Ther. 2018;34(5):410–5. Epub 2018/05/31. 10.1089/jop.2017.0109 . 29812993

[28] Kanada F, Takamura Y, Miyake S, Kamata K, Inami M, Inatani M, et al Histone acetyltransferase and Polo-like kinase 3 inhibitors prevent rat galactose-induced cataract. Sci Rep. 2019;9(1):20085 Epub 2019/12/29. 10.1038/s41598-019-56414-x . 31882756 PMC6934598

[29] Huttenlocher A, Werb Z, Tremble P, Huhtala P, Rosenberg L, Damsky CH. Decorin regulates collagenase gene expression in fibroblasts adhering to vitronectin. Matrix Biol. 1996;15(4):239–50. Epub 1996/09/01. 10.1016/s0945-053x(96)90115-8 . 8892224

[30] Schonherr E, Schaefer L, O’Connell BC, Kresse H. Matrix metalloproteinase expression by endothelial cells in collagen lattices changes during co-culture with fibroblasts and upon induction of decorin expression. J Cell Physiol. 2001;187(1):37–47. Epub 2001/03/10. 10.1002/1097-4652(2001)9999:9999<::AID-JCP1048>3.0.CO;2-W . 11241347

[31] Grant DS, Yenisey C, Rose RW, Tootell M, Santra M, Iozzo RV. Decorin suppresses tumor cell-mediated angiogenesis. Oncogene. 2002;21(31):4765–77. Epub 2002/07/09. 10.1038/sj.onc.1205595 . 12101415

[32] Schonherr E, Sunderkotter C, Schaefer L, Thanos S, Grassel S, Oldberg A, et al Decorin deficiency leads to impaired angiogenesis in injured mouse cornea. J Vasc Res. 2004;41(6):499–508. Epub 2004/11/06. 10.1159/000081806 . 15528932

[33] Chui A, Murthi P, Gunatillake T, Brennecke SP, Ignjatovic V, Monagle PT, et al Altered decorin leads to disrupted endothelial cell function: a possible mechanism in the pathogenesis of fetal growth restriction? Placenta. 2014;35(8):596–605. Epub 2014/06/21. 10.1016/j.placenta.2014.05.009 . 24947404

[34] Jarvelainen H, Sainio A, Wight TN. Pivotal role for decorin in angiogenesis. Matrix Biol. 2015;43:15–26. Epub 2015/02/11. 10.1016/j.matbio.2015.01.023 . 25661523 PMC4560244

[35] Noh KM, Hwang JY, Follenzi A, Athanasiadou R, Miyawaki T, Greally JM, et al Repressor element-1 silencing transcription factor (REST)-dependent epigenetic remodeling is critical to ischemia-induced neuronal death. Proc Natl Acad Sci U S A. 2012;109(16):E962–71. Epub 2012/03/01. 10.1073/pnas.1121568109 . 22371606 PMC3341013

[36] Borrelli E, Nestler EJ, Allis CD, Sassone-Corsi P. Decoding the epigenetic language of neuronal plasticity. Neuron. 2008;60(6):961–74. Epub 2008/12/27. 10.1016/j.neuron.2008.10.012 . 19109904 PMC2737473

[37] Heinrich PC, Behrmann I, Haan S, Hermanns HM, Müller-Newen G, Schaper F. Principles of interleukin (IL)-6-type cytokine signalling and its regulation. Biochem J. 2003;374(Pt 1):1–20. Epub 2003/05/30. 10.1042/BJ20030407 . 12773095 PMC1223585

[38] Yang XM, Wang YS, Zhang J, Li Y, Xu JF, Zhu J, et al Role of PI3K/Akt and MEK/ERK in mediating hypoxia-induced expression of HIF-1alpha and VEGF in laser-induced rat choroidal neovascularization. Invest Ophthalmol Vis Sci. 2009;50(4):1873–9. Epub 2008/12/23. 10.1167/iovs.08-2591 . 19098317

[39] Kinsella RJ, Kahari A, Haider S, Zamora J, Proctor G, Spudich G, et al Ensembl BioMarts: a hub for data retrieval across taxonomic space. Database (Oxford). 2011;2011:bar030 Epub 2011/07/26. 10.1093/database/bar030 . 21785142 PMC3170168

[40] Cunningham F, Achuthan P, Akanni W, Allen J, Amode MR, Armean IM, et al Ensembl 2019. Nucleic Acids Res. 2019;47(D1):D745–d51. Epub 2018/11/09. 10.1093/nar/gky1113 . 30407521 PMC6323964

[41] Subramanian A, Tamayo P, Mootha VK, Mukherjee S, Ebert BL, Gillette MA, et al Gene set enrichment analysis: a knowledge-based approach for interpreting genome-wide expression profiles. Proc Natl Acad Sci U S A. 2005;102(43):15545–50. Epub 2005/10/04. 10.1073/pnas.0506580102 . 16199517 PMC1239896

[42] Wong M, Li Y, Li S, Zhang S, Li W, Zhang P, et al Therapeutic Retrobulbar Inhibition of STAT3 Protects Ischemic Retina Ganglion Cells. Mol Neurobiol. 2015;52(3):1364–77. Epub 2014/10/26. 10.1007/s12035-014-8945-9 . 25344318

[43] Valle ML, Dworshak J, Sharma A, Ibrahim AS, Al-Shabrawey M, Sharma S. Inhibition of interleukin-6 trans-signaling prevents inflammation and endothelial barrier disruption in retinal endothelial cells. Exp Eye Res. 2019;178:27–36. Epub 2018/09/22. 10.1016/j.exer.2018.09.009 . 30240585 PMC6361696

[44] Feng S, Yu H, Yu Y, Geng Y, Li D, Yang C, et al Levels of Inflammatory Cytokines IL-1beta, IL-6, IL-8, IL-17A, and TNF-alpha in Aqueous Humour of Patients with Diabetic Retinopathy. J Diabetes Res. 2018;2018:8546423 Epub 2018/06/01. 10.1155/2018/8546423 . 29850610 PMC5904804

[45] Cheng SC, Huang WC, JH SP, Wu YH, Cheng CY. Quercetin Inhibits the Production of IL-1beta-Induced Inflammatory Cytokines and Chemokines in ARPE-19 Cells via the MAPK and NF-kappaB Signaling Pathways. Int J Mol Sci. 2019;20(12). Epub 2019/06/20. 10.3390/ijms20122957 . 31212975 PMC6628093

[46] Luo W, Brouwer C. Pathview: an R/Bioconductor package for pathway-based data integration and visualization. Bioinformatics. 2013;29(14):1830–1. Epub 2013/06/07. 10.1093/bioinformatics/btt285 . 23740750 PMC3702256

[47] Lamb J, Crawford ED, Peck D, Modell JW, Blat IC, Wrobel MJ, et al The Connectivity Map: using gene-expression signatures to connect small molecules, genes, and disease. Science. 2006;313(5795):1929–35. Epub 2006/09/30. 10.1126/science.1132939 . 17008526

[48] Kisselev AF, Goldberg AL. Proteasome inhibitors: from research tools to drug candidates. Chem Biol. 2001;8(8):739–58. Epub 2001/08/22. 10.1016/s1074-5521(01)00056-4 . 11514224

[49] Lai CY, Yeh DW, Lu CH, Liu YL, Huang LR, Kao CY, et al Identification of Thiostrepton as a Novel Inhibitor for Psoriasis-like Inflammation Induced by TLR7-9. J Immunol. 2015;195(8):3912–21. Epub 2015/09/16. 10.4049/jimmunol.1500194 . 26371257

[50] Kwok BH, Koh B, Ndubuisi MI, Elofsson M, Crews CM. The anti-inflammatory natural product parthenolide from the medicinal herb Feverfew directly binds to and inhibits IkappaB kinase. Chem Biol. 2001;8(8):759–66. Epub 2001/08/22. 10.1016/s1074-5521(01)00049-7 . 11514225

[51] Zeng Y, Cao D, Yu H, Zhuang X, Yang D, Hu Y, et al Comprehensive analysis of vitreous chemokines involved in ischemic retinal vein occlusion. Mol Vis. 2019;25:756–65. Epub 2019/12/10. . 31814701 PMC6857774

[52] Ehlken C, Grundel B, Michels D, Junker B, Stahl A, Schlunck G, et al Increased expression of angiogenic and inflammatory proteins in the vitreous of patients with ischemic central retinal vein occlusion. PLoS One. 2015;10(5):e0126859 Epub 2015/05/16. 10.1371/journal.pone.0126859 . 25978399 PMC4433200

[53] Martin G, Conrad D, Cakir B, Schlunck G, Agostini HT. Gene expression profiling in a mouse model of retinal vein occlusion induced by laser treatment reveals a predominant inflammatory and tissue damage response. PLoS One. 2018;13(3):e0191338 Epub 2018/03/13. 10.1371/journal.pone.0191338 . 29529099 PMC5846732

[54] Koch JC, Barski E, Lingor P, Bahr M, Michel U. Plasmids containing NRSE/RE1 sites enhance neurite outgrowth of retinal ganglion cells via sequestration of REST independent of NRSE dsRNA expression. Febs j. 2011;278(18):3472–83. Epub 2011/07/28. 10.1111/j.1742-4658.2011.08269.x . 21790997

[55] Li J, Wang JJ, Peng Q, Chen C, Humphrey MB, Heinecke J, et al Macrophage metalloelastase (MMP-12) deficiency mitigates retinal inflammation and pathological angiogenesis in ischemic retinopathy. PLoS One. 2012;7(12):e52699 Epub 2013/01/04. 10.1371/journal.pone.0052699 . 23285156 PMC3527600

[56] Zhang LL, Mu GG, Ding QS, Li YX, Shi YB, Dai JF, et al Phosphatase and Tensin Homolog (PTEN) Represses Colon Cancer Progression through Inhibiting Paxillin Transcription via PI3K/AKT/NF-κB Pathway. J Biol Chem. 2015;290(24):15018–29. Epub 2015/04/16. 10.1074/jbc.M115.641407 . 25873394 PMC4463446

[57] López-Colomé AM, Lee-Rivera I, Benavides-Hidalgo R, López E. Paxillin: a crossroad in pathological cell migration. J Hematol Oncol. 2017;10(1):50 Epub 2017/02/20. 10.1186/s13045-017-0418-y . 28214467 PMC5316197

[58] Cehofski LJ, Kruse A, Kjaergaard B, Stensballe A, Honore B, Vorum H. Proteins involved in focal adhesion signaling pathways are differentially regulated in experimental branch retinal vein occlusion. Exp Eye Res. 2015;138:87–95. Epub 2015/06/19. 10.1016/j.exer.2015.06.011 . 26086079

